# Case Report: Maculopathy following standard dose intracameral cefuroxime injection during ICL surgery

**DOI:** 10.3389/fopht.2026.1778421

**Published:** 2026-03-05

**Authors:** Zhenlin Lin, Li Huang, Deli Li, Shouchun Yi, Jingning Weng

**Affiliations:** 1 Fuzhou Aier Eye Hospital, Fuzhou, Fujian, China; 2 Ningde Aier Eye Hospital, Ningde, Fujian, China

**Keywords:** ICL surgery, intracameral cefuroxime, intracameral cefuroxime ocular toxic syndrome (ICOTS), macular edema, subretinal fluid, toxic maculopathy

## Abstract

**Purpose:**

To describe two rare occurrences of acute toxic maculopathy, specifically Intracameral Cefuroxime Ocular Toxic Syndrome (ICOTS), following uncomplicated posterior-chamber toric phakic implantable collamer lens (TICL) surgery despite the administration of a standard dosage of cefuroxime.

**Materials and methods:**

This case report identified two patients from a clinical volume of 1,590 eyes treated between 2021 and 2024. Inclusion criteria consisted of patients undergoing bilateral TICL implantation with intracameral cefuroxime who presented with unexpected visual disturbances on postoperative day 1. To isolate cefuroxime as the primary toxic factor, exclusion criteria included systemic comorbidities (such as diabetes mellitus), pre-existing ocular pathologies (including uveitis, macular degeneration, or prior retinal detachment), history of prostaglandin analog use, or baseline structural abnormalities on preoperative optical coherence tomography (OCT). Clinical evaluation included UDVA, BCVA, and SD-OCT imaging.

**Results:**

Two female patients (aged 29 and 32) underwent bilateral TICL surgery with 0.1 mL of 10 g/L intracameral cefuroxime administered at the conclusion of the procedure. On postoperative day 1, both patients presented with unilateral vision loss (BCVA 20/32 and 20/80, respectively) and reported dim or distorted vision. SD-OCT revealed macular edema and subretinal fluid (SRF). Following conventional postoperative treatment with topical steroids and nonsteroidal anti-inflammatory drugs, complete absorption of SRF was achieved within two weeks to one month, and BCVA improved to 20/16 and 20/20, respectively.

**Conclusion:**

While intracameral cefuroxime is a highly effective prophylactic against endophthalmitis, it may cause sporadic toxic maculopathy even at standard doses during ICL surgery. Unlike typical post-cataract inflammatory edema, ICOTS presents acutely on day 1. Surgeons should maintain a high index of suspicion and utilize early OCT imaging for unexpected postoperative visual disturbances.

## Introduction

As a main surgical method for correcting high myopia, the ICL surgery offers a versatile corrective capacity of up to -20D, characterized by high safety profiles and predictable long-term refractive results. In a follow-up of 17,954 ICL implantations performed by three surgeons, only three cases of endophthalmitis were found (1). However, endophthalmitis is the most serious vision-threatening surgical complication and a major, unavoidable medical catastrophe.

Due to the short operation time and minimal damage associated with ICL surgery, the primary clinical method for preventing postoperative infection is the standardized use of topical antibiotics (2). Referring to the measures for preventing endophthalmitis after cataract surgery, the ESCRS guidelines (3) recommend intracameral injection of 0.1mL of 10g/L cefuroxime as a routine procedure at the end of cataract extraction surgery. A Spanish study showed that intracameral injection of cefuroxime sodium after cataract extraction surgery reduced the incidence of endophthalmitis by 15-fold, from 0.59% to 0.039% (4). Therefore, intracameral cefuroxime sodium injection may be used as a method to prevent postoperative ocular infection after ICL surgery. Several studies have confirmed the safety of intracameral cephalosporins (most commonly studied is cefuroxime) in simple and complex cataract surgeries. Specifically, these studies have shown no significant difference in the effects of cephalosporins on endothelial cell density and macular thickness (5). However, recent clinical evidence has increasingly documented a spectrum of severe adverse drug reactions—ranging from anterior segment inflammation and corneal edema to macular edema, serous retinal detachment, macular infarction and hemorrhagic retinal vasculitis—thereby defining the clinical entity known as Intracameral Cefuroxime Ocular Toxic Syndrome (ICOTS) (6). Beyond structural damage, ICOTS is characterized by significant functional deficits, notably impaired rod photoreceptor responses on electroretinography, with the etiology primarily rooted in the administration of supraphysiological concentrations or excessive dosages during intravitreal or subconjunctival delivery (7–10). Nevertheless, instances of toxicity following conventional dosages have been documented, though cases occurring after posterior-chamber phakic ICL implantation remain exceedingly rare. Only one such case of postoperative macular edema has been published to date. The authors of that study suggested that the condition resulted from an interplay between patient susceptibility, post-laser status, and drug exposure, rather than a solitary toxic factor (11).

In this report, we describe two unique cases of macular edema and subretinal fluid following ICL/TICL implantation, identified from a larger clinical volume of 1,590 eyes treated between 2021 and 2024. Despite receiving a standard 0.1 mL dose of 10 g/L intracameral cefuroxime, both patients reported poor and distorted vision on postoperative day 1, with SD-OCT findings (Carl Zeiss Meditec, Dublin, CA, USA) confirming acute macular involvement.

## Materials and methods

Between 2021 and 2024, a total of 1,590 eyes underwent treatment at our center. From this clinical volume, two specific cases of postoperative macular edema were identified. Clinical evaluation included uncorrected distance visual acuity (UDVA), best-corrected visual acuity (BCVA), and fundus examination. Structural changes were documented using Spectral-Domain Optical Coherence Tomography (SD-OCT; Carl Zeiss Meditec, Dublin, CA, USA).

### Inclusion criteria

Patients who underwent bilateral TICL implantation with intracameral cefuroxime.

Presentation of unexpected visual disturbances on postoperative day 1.

### Exclusion criteria

To isolate the drug as the primary toxic factor, patients were excluded based on the following:

Systemic Comorbidities: Presence of systemic diseases, such as diabetes mellitus.

Pre-existing Ocular Pathologies: History of uveitis, age-related macular degeneration, or prior retinal detachment.

Pharmacological Factors: History of using prostaglandin analogs.

Structural Baseline: Baseline structural abnormalities or vitreomacular traction identified on preoperative OCT.

### Case 1

A 29-year-old female underwent bilateral TICL implantation on the same day. Preoperative examinations showed no systemic diseases such as diabetes, and no history of uveitis or any other significant retinal diseases. Preoperative refractive errors were -7.25/-2.25×180° and -7.5/-1.25 ×165°. Biometry was performed using an IOL Master (Carl Zeiss Meditec, Germany). Axial lengths were 26.43mm and 26.30mm, respectively. Best-corrected visual acuity was 20/20 in both eyes. Preoperative ocular examination showed normal anterior segments and fundi bilaterally. The refractive error and keratometry values remained stable over the past 12 months compared to previous data. A Toric PIOL (Visian ICL with Centraflow; STAAR Surgical) was selected to achieve a postoperative plano refractive state in both eyes. The surgery lasted 13 minutes with no complications. At the end of the surgery, 0.1mL of 10g/L cefuroxime sodium injection was injected into the anterior chamber.

**Figure 1:**
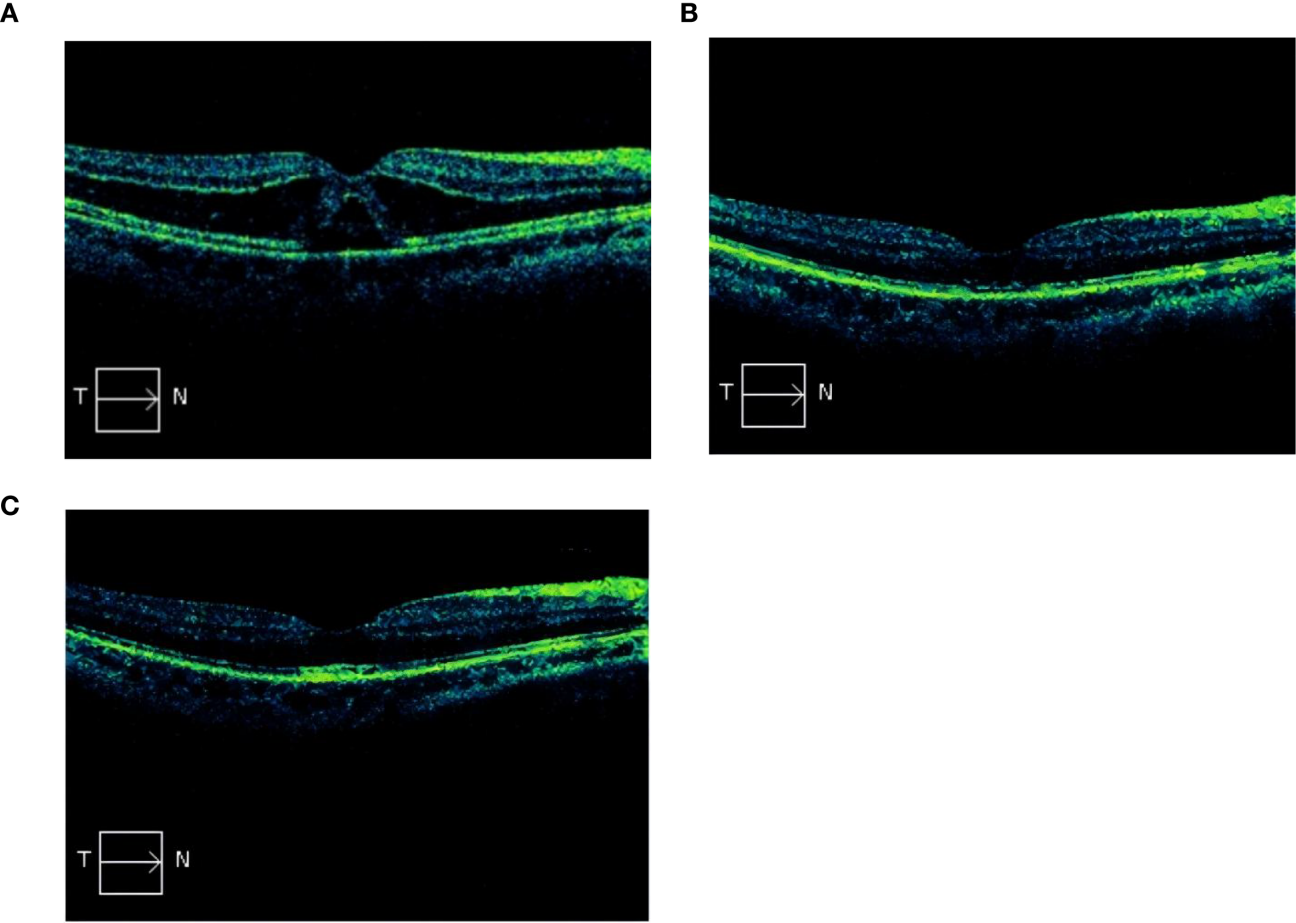
**(A)** Macular edema, mainly manifesting in the outer nuclear layer with perimacular SRF, on Postoperative Day 1. **(B)** At 1 week postoperatively, macular edema and subretinal fluid have been absorbed, central foveal thickness has returned to normal, and the ellipsoid zone is discontinuous. **(C)** At 1 month postoperatively, central foveal thickness has returned to normal, with minimal residual hyper-reflective material remaining after fluid absorption.

On postoperative day 1, the patient’s right eye UDVA and BCVA were 20/32, while the left eye had 20/20 vision with clarity. No significant signs of inflammation or abnormality were observed in the anterior segment or vitreous of the right eye. Fundus examination showed macular edema, and no obvious abnormalities in the peripheral retina. Immediate SD-OCT scanning revealed macular edema with extensive SRF around the macula and optic disc area (**Figure 1A**). The central foveal thickness (CFT) was 648 μm. No obvious vitreomacular traction was found. Topical prednisolone acetate eye drops and pranoprofen eye drops were used four times daily for 1 month. At 1 week postoperatively, the right eye BCVA was 20/32, the ellipsoid zone was discontinuous, and the CFT was reduced to 207 μm (**Figure 1B**). At the 1-month and 3-month follow-up visits, the right eye BCVA was recover to 20/16, central foveal thickness has returned to normal, with minimal residual hyper-reflective material remaining after fluid absorption. The CFT was 205 μm at 1 month postoperatively (**Figure 1C**).

### Case 2

A 32-year-old female underwent bilateral TICL surgery on the same day. She had no systemic diseases and no significant ocular history. Preoperative refractive errors were -9.0/-1.75×5° and -10.75/-1.0×165°. The right eye BCVA was 20/25, and the left eye was 20/20. Biometry was performed using an IOL Master (Carl Zeiss Meditec, Germany). Axial lengths were 26.91mm and 27.43mm, respectively. Preoperative ocular examination showed normal findings. The surgery lasted 15 minutes with no complications. At the end of the surgery, 0.1 mL of 10g/L cefuroxime solution was injected into the anterior chamber.

**Figure 2:**
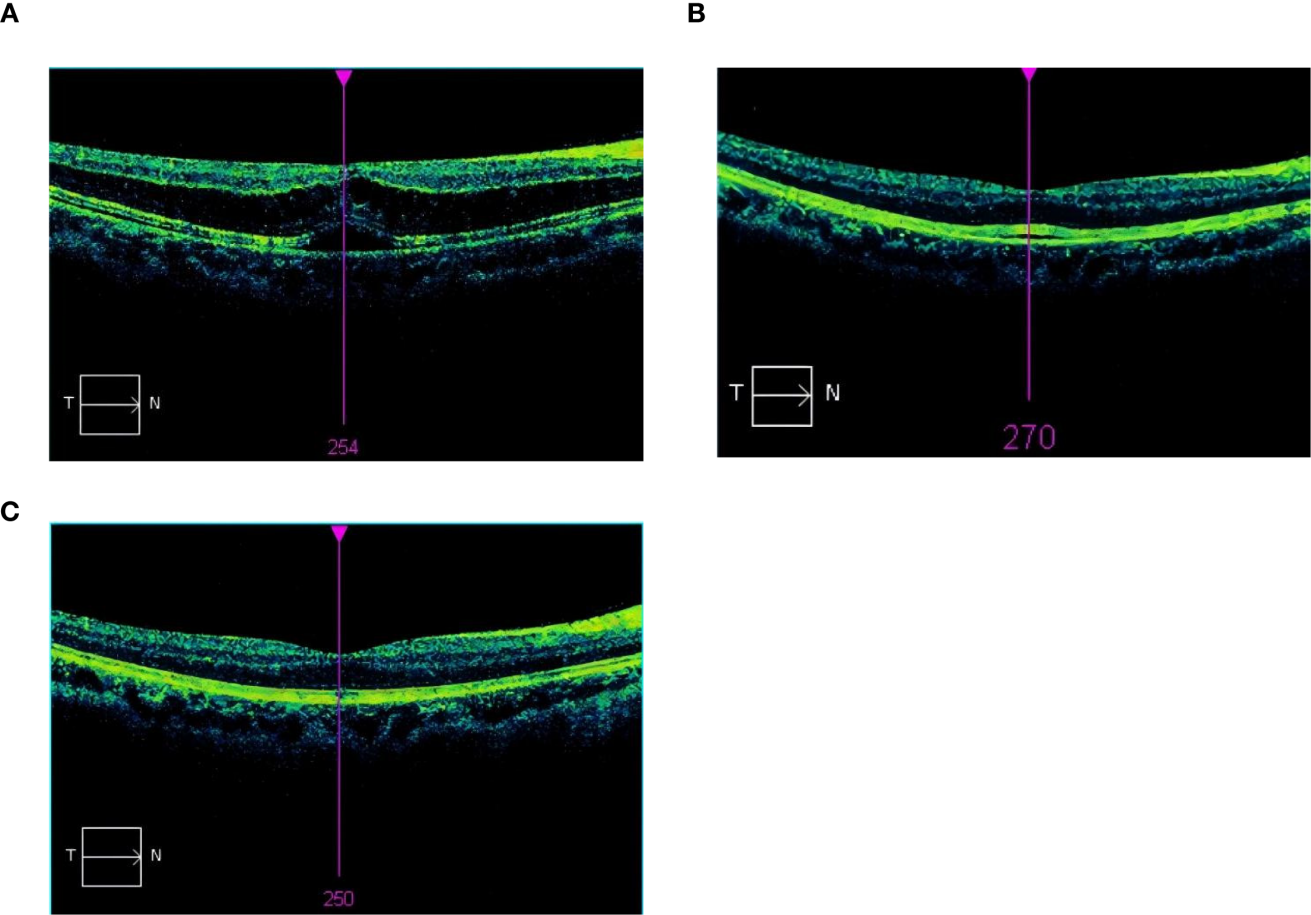
**(A)** Macular edema, mainly manifesting in the outer nuclear layer with perimacular SRF, on Postoperative Day 1. **(B)** At 4 days postoperatively, macular edema and subretinal fluid have partially been absorbed, with a small amount of residual fluid remaining. **(C)** At 2 weeks postoperatively, central foveal thickness has returned to normal.

On postoperative day 1, the patient’s right eye UDVA and BCVA were 20/80, and the left eye UDVA was 20/25. No significant signs of inflammation or abnormality were observed in the anterior segment or vitreous. Fundus examination showed posterior pole retinal pigmentary changes. Immediate SD-OCT scanning revealed macular edema, with shallow SRF in the macula. No vitreomacular traction was found (**Figure 2A**). The CFT was 422 μm. Topical prednisolone acetate eye drops and pranoprofen eye drops were used four times daily for 1 month. At 4 days postoperatively, the right eye BCVA was 20/32, the ellipsoid zone was thickened, a small amount of fluid was visible beneath the OS/RPE layer, and the CFT was reduced to 273 μm (**Figure 2B**). At the 2-week follow-up visit, the right eye BCVA was 20/20, central foveal thickness has returned to normal, and the CFT was 271 μm (**Figure 2C**).

## Discussion

Currently, macular edema (ME) following phakic intraocular lens implantation, such as ICL surgery, remains an exceedingly rare clinical event. Traditionally, sporadic instances of postoperative ME have been attributed to surgical inflammation (11). In highly myopic eyes, structural alterations—including fragility of the retinal pigment epithelium (RPE) and Bruch’s membrane—are thought to increase susceptibility to inflammatory insults by compromising the integrity of the blood-retinal barrier (12). While macular edema is common after cataract surgery as Irvine-Gass syndrome (IGS), it is extremely rare following ICL implantation. Furthermore, the clinical profile of IGS differs significantly from the cases of macular edema described in our reports. These two conditions differ significantly in timing, morphology, and cause. IGS is an inflammatory response that typically peaks 4–6 weeks postoperatively. It is characterized by cystoid macular edema in the inner retinal layers and a “petaloid” leakage pattern on fluorescein angiography. This syndrome is often triggered by surgical trauma, such as posterior capsule rupture or vitreous losses (13). In contrast, our cases represent ICOTS, which is an acute, early-onset toxic reaction. Unlike IGS, this condition manifests even after uncomplicated surgeries with an intact crystalline lens. On OCT, ICOTS shows a distinct “schisis-like” appearance of the outer nuclear layer and extensive subretinal fluid (SRF) (14). This toxicity is likely caused by cephalosporins diffusing to the posterior segment via the zonular-ciliary pathway. While IGS is driven by a prostaglandin-mediated breakdown of the inner blood-retinal barrier, the morphological signature of ICOTS points toward a direct toxic effect on the RPE-photoreceptor complex and Müller cells, likely resulting from the posterior diffusion of cephalosporins through the zonular-ciliary pathway (15, 16). The patients in this report were systemically healthy with no risk factors beyond a tigroid fundus. Preoperatively, the patient in Case 2 exhibited a BCVA of 20/25 in the right eye, deemed consistent with high myopic refractive status; a baseline OCT scan showed no structural abnormalities. Following the incident, OCT imaging demonstrated edema in the outer nuclear layer and caused extensive SRF, which is inconsistent with the imaging findings of Irvine-Gass syndrome. Cefuroxime diffusion to the posterior segment is considered to cause retinal toxicity, leading to vision loss.

The European Society of Cataract and Refractive Surgeons (ESCRS) was the first to recommend cefuroxime injection into the anterior chamber as a routine measure to prevent postoperative infection. However, adverse drug reactions have still been reported in clinical practice, mainly manifested as macular edema and serous retinal detachment. However, according to current literature, retinal detachment surgery, high myopia, age-related macular degeneration, or prostaglandin analogs would not increase the risk of ME after surgery (17).

Drug-induced retinal toxicity can occur with systemic medication, intravitreal drug injection, or topical medication. Researches indicate the distribution and clearance of drugs in the vitreous are influenced by multiple factors, including the ionic properties of the drug, molecular weight, surgical status, and the impact of ocular inflammation (18). Anionic drugs like cephalosporins primarily undergo clearance more rapidly across the blood retinal barrier via the posterior route and exit the eye via uveal blood flow. This is facilitated by active transport by the retinal (19, 20). Therefore, the diffusion of drugs within the vitreous body is difficult to predict, and even at low doses of cefuroxime, it can to some extent explain interindividual variability in sensitivity (21). That despite the blood-ocular barrier, the retina remains susceptible to the toxic effects of systemic drugs, leading to functional impairment and retinal degeneration. These toxicities can be categorized as damage to the retinal pigment epithelium (RPE) and photoreceptor complex, vascular damage, damage to ganglion cells or the optic nerve, macular edema, crystalline retinopathy, uveitis, and changes in color vision and electroretinography (ERG) results. In one study of 9118 cataract surgeries combined with an intracameral injection of cephalosporin (cefazolin 1mg/0.1 ml or cefuroxime 1mg/0.1 ml) at the end of surgery, the probability of postoperative macular edema was 0.88% (22). However, the exact mechanism of cefuroxime toxicity is unclear. Given that macular edema primarily occurs in the outer nuclear layer of the patients in our report, this suggests specific drug sensitivity in the photoreceptors or retinal pigment epithelium (RPE) (23). In an animal model, RPE layer drug sensitivity was observed after high-dose intravitreal antibiotic injection, and fluorescein angiography showed diffuse leakage of retinal vessels, which also suggests that this may be caused by the breakdown of the blood-retinal barrier, and the significant edema of the outer retinal layers may be secondary to abnormal choroidal perfusion. ERG results from animal experiments and clinical observations also suggest that cefuroxime is toxic to the retina and may affect Müller cell function (15, 24). Furthermore, studies have shown that the patterns, morphological characteristics, and thickness of macular edema caused by different diseases have potential pathological differences, which can be initially differentiated by macular OCT examination (25).

Interestingly, a notable observation in our case report is that although both patients underwent bilateral surgery with identical drug administration, only one eye was affected. We propose that this unilateral presentation may stem from subtle physiological asymmetries between the two eyes. Potential factors include localized blood-retinal barrier (BRB) fragility, or minor differences in choroidal circulation and RPE pump function. Additionally, micro-variations during the surgical process—such as transient fluctuations in intraocular pressure or slight discrepancies in drug concentration—could explain why only one eye reached the threshold for toxic injury. These factors likely allowed cefuroxime to reach the posterior segment via the zonular–ciliary pathway, leading to a toxic insult of the RPE-photoreceptor complex and subsequent vision loss.

Although in most cases, the acute exudation leading to macular detachment appears to be transient and this complication is mostly self-limiting, there are still reports of cases where excessive use of cefuroxime sodium injection resulted in persistent damage to the outer retinal structures, such as photoreceptor damage, macular retinal thinning, and subretinal deposits, ultimately leading to vision loss, even after complete resolution of macular edema (26). It is worth noting that the two cases reported in this article are milder than the acute serous retinal detachment (SRD) caused by cefuroxime sodium injection into the anterior chamber during cataract surgery, which has been previously reported. The main manifestation in our patients was SRF. This is likely due to their younger age, shorter surgical duration, minimal trauma, and the retention of the lens. In the two cases we reported, following conventional anti-inflammatory treatment for macular edema, we observed absorption of SRF, and full recovery of vision within 1 month. It is worth noting, however, that the outer layer structure showed changes on OCT in short-term follow-up, suggesting potential residual photoreceptor involvement. Although these two case reports cannot prove that drug-induced maculopathy is self-limiting, they should alert surgeons to actively examine patients with postoperative visual abnormalities for early detection and close follow-up. Furthermore, this report has limitations, as we did not perform electrophysiology (ERG), perimetry, and OCT angiography (OCTA) due to resource constraints to fully assess functional toxicity and microvascular changes, nor can we confirm whether there will be long-term susceptibility to retinal diseases or further lesions.

## Conclusion

While intracameral injection of standard-dose (10 g/L) cefuroxime sodium is a highly effective and generally safe measure for preventing endophthalmitis, it can lead to rare cases of toxic maculopathy following ICL surgery. These cases typically present on postoperative day 1 with macular edema and subretinal fluid. Unlike the acute serous retinal detachment often seen in cataract-related toxicity, the manifestations in ICL patients may be milder due to their younger age and the retention of the crystalline lens. In the reported cases, conventional treatment with topical steroids and NSAIDs resulted in the complete absorption of fluid and recovery of vision within one month (27). Surgeons should maintain a high index of suspicion and perform early OCT imaging for any patient presenting with unexpected postoperative visual disturbances.

## Data Availability

The raw data supporting the conclusions of this article will be made available by the authors, without undue reservation.
